# Quality standard of traditional Chinese medicines: comparison between European Pharmacopoeia and Chinese Pharmacopoeia and recent advances

**DOI:** 10.1186/s13020-020-00357-3

**Published:** 2020-07-28

**Authors:** Fong Leong, Xue Hua, Mei Wang, Tongkai Chen, Yuelin Song, Pengfei Tu, Xiao-Jia Chen

**Affiliations:** 1 State Key Laboratory of Quality Research in Chinese Medicine, Institute of Chinese Medical Sciences, University of Macau, Avenida da Universidade, Taipa, Macao People’s Republic of China; 2 LU-European Center for Chinese Medicine and Natural Compounds, Institute of Biology, Leiden University, Sylviusweg72, 2333BE Leiden, The Netherlands; 3 Science and Technology Innovation Center, Guangzhou University of Chinese Medicine, Guangzhou, 510405 China; 4 Modern Research Center for Traditional Chinese Medicine, School of Chinese Materia Medica, Beijing University of Chinese Medicine, Beijing, 100029 China; 5 State Key Laboratory of Natural and Biomimetic Drugs, School of Pharmaceutical Sciences, Peking University, Beijing, 100191 China

**Keywords:** Traditional Chinese medicine, European Pharmacopoeia, Chinese Pharmacopoeia, Quality standard

## Abstract

Traditional Chinese medicine (TCM) are becoming more and more popular all over the world. However, quality issues of TCM may lead to medical incidents in practice and therefore quality control is essential to TCM. In this review, the state of TCM in European Pharmacopoeia are compared with that in Chinese Pharmacopoeia, and herbal drugs that are not considered as TCM and not elaborated by TCM working party at European Directorate for the Quality of Medicines & Health Care (EDQM) but present in both European Pharmacopoeia and Chinese Pharmacopoeias are also discussed. Different aspects in quality control of TCM including origins, identification, tests and assays, as well as sample preparation, marker selection and TCM processing are covered to address the importance of establishing comprehensive quality standard of TCM. Furthermore, advanced analytical techniques for quality control and standard establishment of TCM are also reviewed.

**Electronic supplementary material:**

The online version of this article (10.1186/s13020-020-00357-3) contains supplementary material, which is available to authorized users.

## Background

Application of herbal drugs or herbal therapies could be dated back since ancient times of human history and it is still being practiced in many places all over the world [1]. Among the herbal drugs applied worldwide, traditional Chinese medicines (TCM) is a large group of plants, animals or minerals applied in remedies following the medical principles developed in ancient China. Nowadays, with system scientific research and modern production technologies, more and more people worldwide have had traditional therapies including TCM in daily life for many reasons [2, 3]. According to “WHO traditional medicine strategy: 2014–2023”, over 100 million Europeans have tried traditional and complementary medicine products and healthcare services, one fifth of the consumers are reported to be regular users. The same phenomenon has also been found in Africa, Asia, Australia and North America with an estimated output of Chinese materia medica to be US$83.1 billion in 2012 [4]. With such amount of consumption worldwide, problems associated with herbal drugs and TCM application have been increasingly reported. For example, a very early report showed that young women applied slimming regimen consisted of *Stephania tetrandra* and *Magnolia officinalis* resulted in renal fibrosis due to the misuse of *Aristolochia fangchi* as *Stephania tetrandra* [5, 6]. It was also reported that exposure to aristolochic acids and their derivatives in herbal drugs (such as *Aristolochia manshuriensis*) could give rise to kidney failure and hepatocellular carcinomas [7]. Medical incidents of herbal drugs treatments are highly related to the quality of herbal drugs, which might due to adulterants, contaminations (such as pesticides and heavy metals), and might also because of the wrong botanical parts used during treatment. Furthermore, incidents may also happen due to processing of herbal drugs, which may involve change in the content of active components and even transforming components in herbal drugs into toxic chemicals. In a recent market study conducted in UK, of the 211 samples investigated, 20 unofficial species were found and 17 samples were detected to have different degree of contamination with other impurities, including other plants, stones and earth etc. [8]. Thus, it is very important to monitor the quality of herbal drugs and TCM.

By realizing the above issue, the Chinese government started to establish the Chinese Pharmacopoeia (ChP) in 1950, and 65 medicaments from plant origins, oils and fats were included in the first edition of ChP (1953 edition). Then, in the second edition (1963 edition) of ChP, TCM was officially organized in Volume I and it was separated from chemical drugs since then. Through years of revisions, the 2020 edition of ChP is the 11th edition of ChP containing 2711 monographs of TCM. On the other hand, European Directorate for the Quality of Medicines & Health Care (EDQM) has started to work on quality control monographs for herbal drugs in European Pharmacopoeia (EP) since 1997. But it was not until 2005, a working program for TCM had been started. The objective of the program is to construct TCM monographs in EP according to the EP’s herbal drugs quality control principles. A special Working Party on TCM constituted by a group of experts and specialists from Europe been formed since 2008 and later on scientists from Asia also joined this working group, to work on a list of TCM candidates with a scheduled working progress [9, 10]. The construction of TCM monographs in EP is on the basis of ChP following the principles, style and technical guideline of EP, with consideration of information in WHO monographs and Hong Kong Chinese Materia Medica Standard [10]. Up until EP10.2 (published in January, 2020), there are 73 herbal drugs considered as TCM in EP according to EP chapter “5.22. Names of herbal drugs used in traditional Chinese medicine”, other herbal drugs such as Ginkgo leaf and St. John’s wort are still considered to be European herbal drugs in EP because of their long history of application in Europe. Since the quality control systems of herbal drugs in Europe and China are different, monographs of TCM are different in the two pharmacopoeias as well. Additional file 1: Table S1 has compared the origin, identification markers, tests and assays of TCM and other European herbal drugs in both pharmacopoeias, differences are found in terms of the botanic origin, quality control markers and methods described in the two pharmacopoeias. Moreover, with more and more studies on TCM quality, new findings of sophisticated analytical techniques have been reported, which may be beneficial in constructing TCM quality control monographs.

Therefore the objectives of this review is to summarize and discuss the differences between the TCM monographs in EP 10th edition (data updated to supplement 10.2, published in January, 2020) and ChP 2020 edition to emphasize the state of TCM monographs in the EP. Note that TCM in this review refer to the 73 herbal drugs considered as TCM in both pharmacopoeias in order to avoid confusions, other examples out of the 73 TCM are given as herbal drugs in general. Furthermore, some advanced analytical techniques for quality standard of herbal drugs and TCM are also discussed to show the progress of TCM quality control.

## Comparison of TCM monographs between EP and ChP

### Origins

Herbal drugs and most of TCM materia medica are derived from plants, thus the origin of TCM usually consist of their botanical sources and medicinal parts. In both pharmacopoeias, origin of the herbal drugs including botanical sources, medicinal parts and their status is specifically stated in the “Definition” section (EP) or at the beginning of each monograph (ChP). A comparison of TCM with different botanical sources and/or medicinal parts is summarized in Table 1. From the table, the most apparent difference is the frequent inclusion of multi-species and/or subspecies for one TCM in ChP. Although EP might also include several botanical origins for one TCM, such as Akebiae Caulis, Coptidis Rhizoma and Piperis Longi Fructus, such cases are not found as frequently as those in ChP. Inclusion of multiple species could be dated back in ancient practice of TCM for many reasons, which may due to different practice between regions and doctors, plant distribution between places, substitution of one species to another, or revision in prescription over time etc. However, significant differences in the chemical profiles may exist between different species of TCM, posing possible quality issues in TCM application. For example, there are three botanical sources of Coptidis Rhizoma stated in both EP and ChP: *Coptis chinensis*, *Coptis deltoidea* and *Coptis teeta*. It was shown that the three species had significant differences in their phytochemical profile, particularly with regard to the pharmacologically active alkaloids [11–13]. Another example is Uncariae Ramulus cum Uncis, EP states that its botanical source is *Uncaria rhynchophylla*, but there are five botanical sources in ChP: *Uncaria rhynchophylla*, *Uncaria macrophylla*, *Uncaria hirsute*, *Uncaria sinensis* and *Uncaria sessilifructus*. The genetic analysis based on rDNA ITS sequences showed that *Uncaria rhynchophylla*, *Uncaria sinensis* and *Uncaria hirsute* were closely related to each other but were far away from *Uncaria macrophylla* and *Uncaria sessilifructus* [14]. The chromatographic fingerprint showed that significant difference could be observed among the five *Uncaria* species, yet there were also some similarities between each other [14, 15]. As mentioned above, there are reasons to include several species and subspecies for one TCM in the monographs, but it brings more difficulty and challenges for the quality control and pharmacological study.

**Table 1:** Comparison of herbal drugs with different botanical source and/or medicinal parts in European Pharmacopoeia and Chinese Pharmacopoeia

European Pharmacopoeia			Chinese Pharmacopoeia		
Latin name	Botanical origins	Medicinal parts	Latin name	Botanical origins	Medicinal parts
Traditional Chinese medicines in both EP and ChP					
Akebiae Caulis	*Akebia quinata* (Houtt.) Decne. or *Akebia trifoliate* (Thunb.) Koidz. or mixture of the 2 species	Stem	Akebiae Caulis	*Akebia quinata* (Thunb.) Decne., *Akebia trifoLiata* (Thunb.)Koidz., or *Akebia trifoliata* (Thunb.) Koidz. var. *australis* (Diels) Rehd.	Lianoid stem
Amomi Fructus	*Amomum villosum* Lour. or *Amomum longiligulare* T. L. Wu	Ripe fruit	Amomi Fructus	*Amomum villosum* Lour., *Amomum villosum* Lour. var. *xanthioides* T. L. Wu et Senjen or *Amomum longiligulare* T. L. Wu	Ripe fruit
Andrographis Herba	*Andrographis paniculata* (Burm.f.) Nees.	Flowering and/or fruit-bearing aerial parts	Andrographis Herba	*Andrographis paniculata* (Burm. f.) Nees	Aerial part
Angelicae Dahuricae Radix	*Angelica dahurica* (Hoffm.) Benth. & Hook. f. ex Franch. & Sav.	Root	Angelicae Dahuricae Radix	*Angelica dahurica* (Fisch. ex Hoffm.) Benth. et Hook. f. or *Angelica dahurica* (Fisch. ex Hoffm.) Benth. et Hook. f. var. formosana (Boiss.) Shan et Yuan	Root
Astragali Mongholici Radix	*Astragalus mongholicus* Bunge	Root	Astragali Radix	*Astragalus membranaceus* (Fisch.) Bge. var. *mongholicus* (Bge.) Hsiao or *Astragalus membranaceus* (Fisch.) Bge.	Root
Clematidis Armandii Caulis	*Clematis armandii* Franch.	Stem	Clematidis Armandii Caulis	*Clematis armandii* Franch. or *Clematis montana* Buch.-Ham.	Lianoid stem
Codonopsis Radix	*Codonopsis pilosula* (Franch.) Nannf.	Root	Codonopsis Radix	*Codonopsis pilosula* (Franch.) Nannf., *Codonopsis pilosula* Nannf. var. *modesta* (Nannf.) L. T.Shen or *Codonopsis tangshen* Oliv.	Root
Ephedrae Herba	*Ephedra sinica* Stapf, *Ephedra intermedia* Schrenk et C.A.Mey. or *Ephedra equisetina* Bunge	Aerial parts	Ephedrae Herba	*Ephedra sinica* Stapf, *Ephedra intermedia* Schrenk et C.A. Mey. or *Ephedra equisetina* Bge.	Herbaceous stem
Fraxini Chinensis Cortex	*Fraxinus chinensis* subsp. *rhynchophylla* (Hance) A.E.Murray (syn. *Fraxinus rhynchophylla* Hance)	Branch or trunk bark	Fraxini Cortex	*Fraxinus rhynchophylla* Hance, *Fraxinus chinensis* Roxb, *Fraxinus szaboana* Lingelsh. or *Fraxinus stylosa* Lingelsh.	Branch or stem bark
Magnoliae Biondii Flos Immaturus	*Magnolia biondii* Pamp. (syn. *Yulania biondii* (Pamp.) D.L.Fu)	Flower bud	Magnoliae Flos	*Magnolia biondii* Pamp., *Magnolia denudata* Desr. or *Magnolia sprengeri* Pamp.	Flower bud
Magnoliae officinalis cortex	*Magnolia officinalis* Rehder. et E.H. Wilson.	Stem and branch bark	Magnoliae Officinalis Cortex	*Magnolia officinalis* Rehd. et Wils. or *Magnolia officinalis* Rehd. et Wils. var. *biloba* Rehd. et Wils.	Stem bark
Magnoliae officinalis flos	*Magnolia officinalis* Rehder et E.H. Wilson.	Unopened flower	Magnoliae Officinalis Flos	*Magnolia officinalis* Reld. et Wils. or *Magnolia officinalis* Rehd. et Wils. var. *biloba* Rehd. et Wils.	Flower bud
Notoginseng Radix	*Panax notoginseng* (Burkill) F.H.Chen [*Panax pseudoginseng* var. *notoginseng* (Burkill) G.Hoo and C.L.Tseng]	Root	Notoginseng Radix et Rhizoma	*Panax notoginseng* (Burk.) F. H. Chen	Root and rhizome
Piperis Longi Fructus	*Piper longum* L. or *Piper retrofractum* Vahl (syn. *P. chaba* Hunter and *P. officinarum* (Miq.) C. DC.) or a mixture of both species	Ripe or nearly ripe fruiting spikes	Piperis Longi Fructus	*Piper longum* L.	Ripe or nearly ripe fruit-spike
Sanguisorbae Radix	*Sanguisorba officinalis* L.	Underground parts	Sanguisorbae Radix	*Sanguisorba officinalis* L. or *Sanguisorba officinalis* L. var. *longifolia* (Bert.) Yu et Li	Root
Uncariae Rhynchophyllae Ramulus cum Uncis	*Uncaria rhynchophylla* (Miq.) Miq. ex Havil.	Branch or stem with hooks	Uncariae Ramulus cum Uncis	*Uncaria rhynchophylla* (Miq.) Jacks., *Uncaria macrophylla* Wall., *Uncaria hirsuta* Havil., *Uncaria sinensis* (Oliv.) Havil., *Uncaria sessilifructus* Roxb.	Hook-bearing branch
Zanthoxyli Bungeani Pericarpium	*Zanthoxylum bungeanum* Maxim.	Pericarp of the ripe fruit	Zanthoxyli Pericarpium	*Zanthoxylum schinifolium* Sieb. et Zucc. or *Zanthoxylum bungeanum* Maxim.	Pericarp of the ripe fruit
Other herbal drugs in both EP and ChP					
Belladonnae Folium	*Atropa belladonna* L.	Leaf and flowering tops	Belladonnae Herba	*Atropa belladonna* L.	Herb
Centellae Asiaticae Herba	*Centella asiatica* (L.) Urb.	Aerial parts	Centellae Herba	*Centella asiatica* (L.) Urb.	Herb
Chelidonii Herba	*Chelidonium majus* L.	Flowering aerial parts	Chelidonii Herba	*Chelidonium majus* L.	Herb
Eleutherococci Radix	*Eleutherococcus senticosus* (Rupr. et Maxim.) Maxim.	Underground organs	Acanthopanacis Senticosi Radix et Rhizoma seu Caulis	*Acanthopanax senticosus* (Rupr. et Maxim.) Harms	Root, rhizome or stem
Ginseng Radix	*Panax ginseng* C. A. Mey.	Root	Ginseng Radix et Rhizoma	*Panax ginseng* C. A. Mey.	Root and rhizome
Hyperici Herba	*Hypericum perforatum* L.	Flowering tops	Hyperici Perforati Herba	*Hypericum perforatum* L.	Flowering aerial parts
Liquiritiae Radix	*Glycyrrhiza glabra* L. and/or *Glycyrrhiza inflata* Bat. and/or *Glycyrrhiza uralensis* Fisch.	Root and stolons	Glycyrrhizae Radix et Rhizoma	*Glycyrrhiza uralensis* Fisch., *Glycyrrhiza inflata* Bat. or *Glycyrrhiza glabra* L.	Root and rhizome
Polygalae Radix	*Polygala senega* L. or *Polygala tenuifolia* Willd.	Root and/or root crown	Polygalae Radix	*Polygala tenuifolia* Willd. or *Polygala sibirica* L.	Root
Polygoni Avicularis Herba	*Polygonum aviculare* L. s.l.	Flowering aerial parts	Polygoni Avicularis Herba	*Polygonum aviculare* L.	Aerial parts
Rhei Radix	*Rheum palmatum* L. or *Rheum officinale* Baillon or hybrids of these two species or of a mixture	Underground parts	Rhei Radix et Rhizoma	*Rheum palmatum* L., *Rheum tanguticum* Maxim. ex Balf., or *Rheum officinale* Baill.	Root and rhizome
Taraxaci Officinalis Herba cum Radice	*Taraxacum officinale* F.H. Wigg.	Aerial and underground parts	Taraxaci Herba	*Taraxacum mongolicum* Hand. -Mazz., *Taraxacum borealisinense* Kitam. or several other species of the same genus	Herb

In addition, Latin synonyms in pharmacopoeias could be an issue for identification of botanical origins of TCM. An analysis showed that at least 16.13% Latin names of TCM in ChP (2010 edition) were not in accordance with Flora of China and the reasons of the issue may include: repeat naming of the same species; synonyms of the families; new definitions of species and families; as well as traditional use of old Latin names [16]. Among the 73 TCM reviewed, 24 entries have Latin synonyms stated in EP, and some other TCM without annotation may also have Latin synonyms. For example, botanical origins of Sinomenii Caulis in ChP include *Sinomenium acutum* (Thunb.) Rehd. et Wils. or *Sinomenium acutum (Thunb.)* Rehd. et Wils. var. *cinereum* Rehd. et Wils. But in fact, the latter is synonym of the former, and the former is the botanical origin stated in EP, thus the botanical origin of Sinomenii Caulis in both pharmacopoeias is actually the same. Therefore, when determining the botanical origins of TCM, Latin synonyms is still an issue that should be addressed.

In the application of TCM, herbs could be applied either as in whole plant, or as in different parts of the plant such as aerial parts, underground parts, root, rhizome, stem, bark, leaf or flower. Furthermore, active constituents and harmful constituents may be varied in different parts of an herb. Therefore, it is very important to specify the medicinal parts in quality standard of TCM. By comparing the stated medicinal parts of TCM between the two pharmacopoeias, most are the same except few such as Ephedrae Herba and Sanguisorbae Radix (Table 1). But the differences in these TCM are small, such as underground parts instead of root, thus little or no influence would be generated under the circumstances.

The above examples imply that it is important to specify the botanical source and the medicinal part of a TCM, because substantial differences may occur in the components and pharmacological effects when the wrong plant or medicinal parts are applied. However, the origin of herbal drugs and TCM stated in pharmacopoeias may be more or less influenced by the species available and the application habit in the region. Therefore, extensive research are required to show that if those differences could be compatible with each other and whether substitution is possible for one to another.

### Identification

TCM identification in EP and ChP have many similarities, but they also have several significant differences in terms of method and marker selections. For a typical TCM monograph, identification includes: macroscopic examination of the herbal drug’s botanical characteristics such as their shape, color or surface texture; microscopic examination of the powder for microstructure inspection of the herbal drug’s tissues and cells; and thin layer chromatography (TLC) for chemical-based identification. All the tests are important in TCM identification because macroscopic and microscopic examination are more convenient for community pharmacies and consumers to easily identify TCM with less sophisticated equipment, and TLC identification is a more accurate and precise method to identify TCM in a more equipped laboratory. Whether to use some of the above tests or all of them depends on whether the method is feasible or has significant meaning in TCM identification, also special identification tests may sometimes be needed for further identification of the TCM from other similar herbs. In EP, TLC analysis of a TCM may be used as both identification and controlling adulterants in the monograph, the method under this situation is described in “Tests” and the method would be cross-referred to “Identiication”. For example, Angelicae Dahuricae Radix, Angelicae Pubescentis Radix and Angelicae Sinensis Radix all incorporate a TLC test to differentiate the TCM with other officinal species of *Angelica*, *Levisticum* and *Ligusticum*, the method is also utilized as TLC identification for these three TCM.

In both pharmacopoeias, TLC is the most important identification method for TCM, because many herbal drugs share very similar features and it is often very hard to distinguish one from another by macroscopic and microscopic identification. Also, TLC is a relatively simple and convenient method for accurate, precise and easily interpreted identification of TCM, compared to techniques such as high performance liquid chromatography (HPLC) and gas chromatography (GC). The general procedures of TLC identification in both pharmacopoeias are the same, but EP and ChP employ different styles to illustrate TLC results. TLC results of EP are usually presented as a schematic box showing the positions of the bands. On the other hand, TLC results of ChP are presented as a simple description: “The spot in the chromatogram obtained with the test solution corresponds in position and colour to the spot in the chromatogram obtained with the reference solution”. In addition, EP and ChP may use quite different TLC method for TCM identification based on the selected marker. For example, TLC identification method for Belamcandae Rhizoma including solid phase (EP: silica gel plate; ChP: polyamide film), mobile phase (EP: glacial acetic acid, cyclohexane and ethyl acetate in 1:20:80; ChP: chloroform, butanone and methanol in 3:1:1) and detection (EP: 254 nm; ChP: 365 nm after visualization with aluminium trichloride solution) are different between EP and ChP, mainly because of the different marker used (EP: irisflorentin and coumarin; ChP: Belamcandae Rhizoma reference drug).

### Test

In both pharmacopoeias, different tests are required to detect different contaminations and adulterants according to the nature of each TCM. EP has a general monograph named “Herbal drugs” and required tests are listed in the monograph, including foreign matter, loss on drying, water, pesticides, heavy metals, total ash, ash insoluble in hydrochloric acid, extractable matter, swelling index, bitterness value, aflatoxin B_1_, ochratoxin A, radioactive contamination and microbial contamination. Each test is cross-referred to other chapters of EP which specify the analytical methods. General requirements of foreign matter and heavy metals are included in this general monograph, and general limits of pesticides and aflatoxin B_1_, are given in the monographs of corresponding analytical methods. ChP also has a general chapter entitled “0212 General principle for inspection of crude drugs and decoction pieces” requiring the tests for the content of water, ash, foreign matters, poisonous ingredients, heavy metals, harmful elements, pesticides residues, aflatoxins, etc., and gives the general limits of the content of water, foreign matter, sulfur dioxide and pesticides. The relevant methods are provided in a series of general chapters under the catalogue “2000 Special methods for traditional Chinese medicines”. Besides the above differences in the general monograph, some other differences are also found when comparing the “Tests” section in TCM monographs between EP and ChP, which should be taken into consideration in quality control of TCM.

In quality assessment of TCM, moisture content is an important issue because inappropriate moisture would facilitate microbes’ growth, resulting in decomposition or toxin generation in TCM. Therefore “Loss on drying” or “Water” is often required in the monograph to determine TCM’s moisture content. However, even though “Water” and “Loss on drying” seem to be very similar, they are different not only in their method, but also what they convey in the monographs. “Loss on drying” determines the weight loss of TCM during specific condition such as heating or vacuum, which may include water and volatile contents in TCM, while “Water” determines only the moisture in TCM. Usually, the result of “Loss on drying” and “Water” will be the same for TCM with no or little volatile substances. But significant difference in the results may happen when applying different methods to determine the TCM containing high volatile content [17, 18]. A comparison of the methods for moisture determination in the two pharmacopoeias is summarized in Table 2. There is only little difference between the methods of “Loss on drying”, but major differences could be found in “Water”. EP has only included toluene distillation in the method, but ChP has also included Karl-Fischer titration, drying in oven, drying under reduced pressure and GC as well. When comparing the frequency of different methods for TCM moisture determination, drying in oven method is the most applied. Among the 73 TCM reviewed, 66 in EP and 51 in ChP employ this method. Furthermore, 7 TCM monographs in EP and 13 in ChP use toluene distillation method, mainly based on the volatile content of the TCM, since EP states that toluene distillation method instead of drying in oven or in vacuo should be carried out for herbal drugs with high essential oil content, ChP also states that drying in oven method should be used for crude drugs with little or no volatile constituents. Table 3 shows the differences in the methods between the two pharmacopoeias with the TCM’s essential oil contents for reference. A typical example would be Atractylodis Macrocephalae Rhizoma, limit for “Water” in EP and ChP are 10% (100 mL/kg) and 15%, respectively. The significant difference in the standard may mainly due to the difference in the methods, because the essential oil content of Atractylodis Macrocephalae Rhizoma should be no less than 9 mL/kg according to EP. Furthermore, parameters of the methods applied in pharmacopoeias may also have impact on the result. EP specifies drying process to be 105 °C usually for certain hours, while ChP requires the sample to be dried until the difference between two successive weighings is not more than 5 mg, thus residual in moisture may affect the result and standard of certain TCM, such as Bupleuri Radix (EP: 5%; ChP: 10%), Isatidis Radix (EP: 9%; ChP: 15%) and Schisandrae Chinensis Fructus (EP: 10%; ChP: 16%) (Additional file 1: Table S1). Therefore, consideration should be taken when using either “Loss on drying” or “Water” for a specific TCM, because different results may be obtained when using different methods to determine the moisture content in TCM.

**Table 2:** Summary of the methods for determination of “loss on drying” and “water” in European Pharmacopoeia and Chinese Pharmacopoeia

European Pharmacopoeia	Chinese Pharmacopoeia
Loss on drying	
Dry the sample under the specified temperature to constant mass (Δm^a^ ≤ 0.5 mg) or for the prescribed time by one of the following procedures and calculate the difference in the mass of the sample before and after drying, expressed as a percentage (m/m): In a desiccator In vacuo In an oven at a specified temperature	Place about 1 g or specified amount of sample in a tared, shallow weighing bottle and dry the sample under 105 °C until constant weight (Δm ≤ 0.3 mg) except as otherwise herein provided. Calculate the loss of mass expressed as per cent Test may also be done with desiccator with temperature control or vacuum
Water	
Distillation with toluene (procedure similar to method 4 in Chinese Pharmacopoeia)	Method 1: Karl-Fischer’s titration Method 2: Drying in the oven (100–105 °C until Δm ≤ 5 mg) Method 3: Drying under reduced pressure (≤ 2.67 kPa at room temperature for 24 h) Method 4: Toluene distillation Method 5: Gas chromatography

^a^ Δm: the difference in the mass of the sample between two consecutive weighings

**Table 3:** Comparison of herbal drugs with different methods for “loss on drying” or “water” content and their essential oil contents in European Pharmacopoeia and Chinese Pharmacopoeia

European Pharmacopoeia				Chinese Pharmacopoeia			
Latin name	Methods	Limits	Contents of essential oil	Latin name	Methods	Limits	Contents of essential oil
Traditional Chinese medicines in both EP and ChP							
Andrographis Herba	LD (105 °C, 2 h)	≤ 10.0%	Not included	Andrographis Herba	Not included	Not included	Not included
Angelicae Dahuricae Radix	LD (105 °C, 2 h)	≤ 12.0%	Not included	Angelicae Dahuricae Radix	Water (toluene distillation)	≤ 14.0%	Not included
Angelicae Pubescentis Radix	LD (105 °C, 2 h)	≤ 10.0%	Not included	Angelicae Pubescentis Radix	Water (toluene distillation)	≤ 10.0%	Not included
Angelicae Sinensis Radix	LD (105 °C, 2 h)	≤ 12.0%	Not included	Angelicae Sinensis Radix	Water (toluene distillation)	≤ 15.0%	≥ 0.4%
Atractylodis Macrocephalae Rhizoma	Water (toluene distillation)	≤ 100 mL/kg	≥ 9 mL/kg	Atractylodis Macrocephalae Rhizoma	Water (100–105 °C until Δm^a^ ≤ 5 mg)	≤ 15.0%	Not included
Aucklandiae Radix	LD (105 °C, 2 h)	≤12.0%	Not included	Aucklandiae Radix	Not included	Not included	Not included
Citri Reticulatae Epicarpium et Mesocarpium	LD (105 °C, 2 h)	≤ 12.0%	Not included	Citri Reticulatae Pericarpium	Water (toluene distillation)	≤ 13.0%	Not included
Eucommiae Cortex	LD (105 °C)	≤ 12.0%	Not included	Eucommiae Cortex	Not included	Not included	Not included
Ligustici Chuanxiong Rhizoma	LD (105 °C, 2 h)	≤ 8.0%	≥ 3.5 mL/kg	Chuanxiong Rhizoma	Water (toluene distillation)	≤ 12.0%	Not included
Ligustici Radix et Rhizoma	LD (105 °C)	≤ 12.0%	≥ 5.0 mL/kg	Ligustici Rhizoma et Radix	Water (toluene distillation)	≤ 10.0%	Not included
Lycii Fructus	LD (105 °C, 2 h)	≤ 11.0%	Not included	Lycii Fructus	Water (80 °C until Δm ≤ 5 mg)	≤ 13.0%	Not included
Magnoliae Biondii Flos Immaturus	Water (toluene distillation)	≤ 100 mL/kg	≥ 14.0 mL/kg	Magnoliae Flos	Water (GC)	≤ 18.0%,	≥ 1.0%
Magnoliae Officinalis Cortex	LD (105 °C, 2 h)	≤ 11.0%	Not included	Magnoliae Officinalis Cortex	Water (toluene distillation)	≤ 15.0%	Not included
Magnoliae Officinalis Flos	LD (105 °C)	≤ 11.0%	Not included	Magnoliae Officinalis Flos	Water (reduced pressure (≤ 2.67 kPa) at room temperature for 24 h)	≤ 10.0%	Not included
Moutan Cortex	LD (105 °C, 2 h)	≤ 11.0%	Not included	Moutan Cortex	Water (toluene distillation)	≤ 13.0%	Not included
Paeoniae Radix Rubra	LD (105 °C, 2 h)	≤ 12.0%	Not included	Paeoniae Radix Rubra	Not included	Not included	Not included
Persicariae Tinctoriae Folium	LD (105 °C, 2 h)	≤ 7.0%	Not included	Polygoni Tinctorii Folium	Not included	Not included	Not included
Piperis Longi Fructus	LD (105 °C, 2 h)	≤ 11.0%	≥ 6.0 mL/kg	Piperis Longi Fructus	Water (toluene distillation)	≤ 11.0%	Not included
Polygoni Orientalis Fructus	LD (105 °C, 2 h)	≤ 12.0%	Not included	Polygoni Orientalis Fructus	Not included	Not included	Not included
Zanthoxyli Bungeani Pericarpium	Water (toluene distillation)	≤ 100 mL/kg	≥ 15 mL/kg	Zanthoxyli Pericarpium	Not included	Not included	≥ 1.5%
Other herbal drugs in both EP and ChP							
Allii Sativi Bulbi Pulvis	LD (105 °C)	≤ 7.0%	Not included	Allii Sativi Bulbus	Not included	Not included	Not included
Anisi Stellati Fructus	Water (toluene distillation)	≤ 100 mL/kg	≥ 70 mL/kg	Anisi Stellati Fructus	Not included	Not included	≥ 4.0%
Belladonnae Folium	Not included	Not included	Not included	Belladonnae Herba	Water (100–105 °C until Δm^a^ ≤ 5 mg)	≤ 13.0%	Not included
Capsici Fructus	LD (105 °C, 2 h)	≤ 11.0%	Not included	Capsici Fructus	Not included	Not included	Not included
Caryophylli Flos	Not included	Not included	≥ 150 mL/kg	Caryophylli Flos	Water (toluene distillation)	≤ 12.0%	Not included
Foeniculi Amari Fructus	Water (toluene distillation)	≤ 100 mL/kg	≥ 40 mg/kg	Foeniculi Fructus	Not included	Not included	≥ 1.5%
Foeniculi Dulcis Fructus	Water (toluene distillation)	≤ 80 mL/kg	≥ 20 mg/kg	Foeniculi Fructus	Not included	Not included	≥ 1.5%
Lini Semen	LD (105 °C, 2 h)	≤ 8.0%	Not included	Lini Semen	Not included	Not included	Not included
Myrrha	LD (105 °C, 2 h)	≤ 15.0%	Not included	Myrrha	Not included	Not included	≥ 4.0% for natura myrrh, ≥ 2.0% for colloidal myrrh

*LD* loss on drying

^a^ Δm: the difference in the mass of the sample between two consecutive weighings

Pesticides are substances used to prevent, destroy or control pest, unwanted plants or animals during the production of herbal drugs. Pesticides may remain in the TCM if inappropriate approach is conducted during production, which will become pesticides residues and could be potential toxins to consumers. Therefore, EP requires pesticide residue test for herbal drugs and it is cross-referred to chapter “2.8.13 Pesticides residues”. A list consisted of 69 pesticides’ limits is included in the chapter, limits of other pesticides are cross-referred to Regulation (EC) No. 396/2005 or calculated by acceptable daily intake amount, body weight and daily dose of the herbal drug. Although there is no specification on the methods, analysis must be validated according to the requirements stated in chapter 2.8.13. In ChP, general chapter “2341 Determination of pesticide residues” specifies GC, GC–mass spectrometry (MS) or LC–MS techniques to determine the pesticide residues in TCM. The chapter includes several categories of pesticides to be determined (organochlorine, organophosphorous, pyrethrin, etc.) and each has a detail description of determination method and a list of pesticides and their retention time, limit of detection etc. as guidance for quality control performers. The limits of 33 pesticides are given in the general chapter “0212 General principle for inspection of crude drugs and decoction pieces”, and special requirements are included for some herbal drugs, such as Astragali Radix, Ginseng Radix et Rhizoma and Glycyrrhizae Radix et Rhizoma (Additional file 1: Table S1).

Heavy metals may accumulate in TCM during production, causing symptoms such as anaemia, pains or organ failure to TCM users, thus heavy metals content should be strictly controlled in TCM [19]. EP chapter “2.4.27 Heavy metals in herbal drugs and herbal drug preparation” has included determination methods for arsenic, cadmium, copper, mercury, nickel and lead. The limits for herbal drugs in EP are as follow: cadmium (≤ 1.0 ppm), lead (≤ 5.0 ppm), mercury (≤ 0.1 ppm), but special requirements may be needed for some herbal drugs such as Lini Semen (cadmium ≤ 0.5 ppm). As for ChP, “Heavy metals and harmful elements” are required only in some monographs (Angelicae Dahuricae Radix, Angelicae Sinensis Radix, Astragali Radix, Gardeniae Fructus, Lycii Fructus, Notoginseng Radix et Rhizoma, Paeoniae Radix Alba, Puerariae Lobatae Radix, Salviae Miltiorrhizae Radix et Rhizoma, etc.) and cross-referred to general chapter “2321 Determination of lead, cadmium, arsenic, mercury and copper”, with limit of each element stated in each monograph (usually as follow: lead ≤ 5 mg/kg, cadmium ≤ 1 mg/kg, arsenic ≤ 2 mg/kg, mercury ≤ 0.2 mg/kg, copper ≤ 20 mg/kg). Atomic absorption spectrometry and inductively coupled plasma (ICP)-MS are introduced in this general chapter as the determination methods of heavy metals content in TCM, which are the mostly applied methods in heavy metal determination nowadays [20]. In addition, different forms or species of heavy metals determine their properties and especially toxicity. For example, the LD_50_ values of different arsenic species in rat are different, the most toxic species would be arsine with LD_50_ of 3.0 mg/kg, but monomethylarsonic acid and dimethylarsinic acid would be considered “non-toxic” with LD_50_ between 700 and 2600 mg/kg [21]. In TCM, heavy metals could also present in the herb in many forms, especially for arsenic and mercury [22–24]. Therefore, it is important to identify the forms of heavy metals in TCM to rationally assess the potential hazard of inorganic impurities in TCM. ChP general chapter “2322 Determination of mercury and arsenic speciation and their valence states” has specified HPLC–ICP-MS to determine the species of arsenic and mercury in TCM. The method is now the most applied method in heavy metal speciation and has been applied in different TCM for heavy metal speciation [25–27]. But still, heavy metal speciation is only required for Cinnabaris (mercuric sulfide) and Realgar (arsenic disulfide) in ChP. Therefore, further research is needed to generate more data and knowledge in this area in order to develop advanced and rational inorganic impurities regulations for TCM.

In TCM production, TCM may be fumigated with sulfur as post-harvest process. Sulfur fumigation could have beneficial effects on TCM including preservation and better appearance but it also generates problems such as sulfur dioxide and heavy metal residues and changes in the chemical profile in TCM [28]. Since sulfur fumigation is an effective, low cost and traditional processing method in TCM production, many TCM crude drugs may go through sulfur fumigation before going to the market, which may cause toxicity to consumers, decrease in TCM quality and give rise to counterfeiting in the market, therefore determination of sulfur dioxide residue is necessary in order to prevent irrational use of sulfur fumigation in TCM production. In comparison, sulfur dioxide residue is not required in EP, but it is required in ChP that limit of sulfur dioxide residue generally do not exceed 150 mg/kg except mineral drugs, and 400 mg/kg for 10 particular TCM including Achyranthis Bidentatae Radix, Atractylodis Macrocephalae Rhizoma, Codonopsis Radix, Dioscoreae Rhizoma, Gastrodiae Rhizoma, Paeoniae Radix Alba and Puerariae Thomsonii Radix, etc. In terms of determination method, ChP general chapter “2331 Determination of residue of sulfur dioxide” employs acid–base titration, GC and ion chromatography to determine the sulfur dioxide residue in TCM, quality control conductor may choose the appropriate method to determine the sulfur dioxide residue in TCM.

When TCM is stored under suitable temperature and humidity conditions for microorganisms, fungi and molds may grow in TCM and generate a large group of secondary metabolic products called mycotoxins. Mycotoxins consist of many categories including aflatoxin, ochratoxin, zearalenone, etc. and have hazardous effects to human body such as hepatic cell and tissue injury, reproductive disorders and diarrhea etc. [29]. Thus, both EP and ChP have included tests to control the mycotoxins level for TCM. Generally, EP chapter “2.8.18 Determination of aflatoxin B_1_ in herbal drugs” and “2.8.22 Determination of ochratoxin A in herbal drugs” require herbal drugs subjected to contamination by aflatoxins B_1_ or ochratoxin A should be tested by a validated method, and give the limit of aflatoxin B_1_ (aflatoxin B_1_ ≤ 2 µg/kg, sum of G_2_, G_1_, B_2_ and B_1_ ≤ 4 µg/kg). Special limit for the two mycotoxins may be required if necessary (e.g. ochratoxin A in Liquiritiae Radix ≤ 20 μg/kg). ChP also requires some herbal drugs to have their mycotoxin content determined, and of the herbal drugs reviewed, Citri Reticulatae Pericarpium, Coicis Semen, Corydalis Rhizoma and Polygalae Radix are required to test their aflatoxins (aflatoxin B_1_ ≤ 5 µg/kg, sum of G_2_, G_1_, B_2_ and B_1_ ≤ 10 µg/kg), Coicis Semen is required to test its zearalenone (≤ 500 µg/kg). For mycotoxins determination, EP applies LC-fluorescence detection to determine the levels of aflatoxins B_1_ and ochratoxin A in TCM, while in ChP general chapter “2351 Determination of mycotoxins”, LC and/or LC–MS are specified to determine the mycotoxins in TCM, including aflatoxins, ochratoxin A, zearalenone, deoxynivalenol, patulin, etc. Additionally, enzyme-linked immunosorbent assay can also be used for aflatoxins determination.

“Extractable matter” in EP or “Extractives” in ChP determines the content of substances in TCM extracts using different solvents such as water, ethanol and ether. However, instead of a requirement in “Test” section, “Extractives” in ChP is separated from “Test” as an individual item in the monographs. Furthermore, of the 73 TCM reviewed, EP only requires 5 TCM to have their extractable matter determined (Acanthopanacis Gracilistyli Cortex, Codonopsis Radix, Dioscoreae Oppositifoliae Rhizoma, Lycii Fructus and Poria), all without assay requirements in the monographs; on the other hand, a total of 50 TCM in ChP need to determine their content of extractives, while the majority of them also have assay quantification in their monographs. The reason of EP not to include extractable matter in certain monographs is because extractable matter determination is useful only to TCM without a component suitable for an assay or TCM used to produce a preparation with a dry residue [30]. The methods used to determine extractable matter or extractives in both pharmacopoeias are very similar: certain amount of TCM is extracted with specific solvent, then the filtrate is evaporated to dryness and the residue is weighed to calculate the percentage of the extracts. However, EP does not have a general chapter for extractable matter, and methods are included only in corresponding monographs with stated limits for particular TCM; ChP on the other hand includes general chapter “2201 Determination of extractives” and three types of extractives including water, ethanol and volatile ether are described.

### Assay

**Fig. 1:**
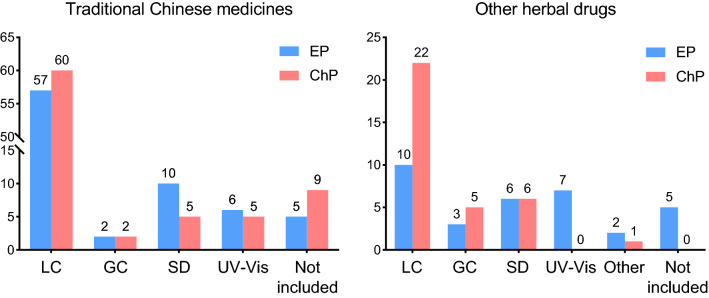
Numbers of analytical methods applied in “Assay” section for herbal drugs recorded in both European Pharmacopoeia and Chinese Pharmacopoeia

Besides TCM identification and different quality tests, one of the most important quality indicators for TCM is the content of active components, which is assessed in “Assay” in both pharmacopoeias. But EP and ChP may apply different techniques to assess the active components of TCM, and sometimes “Assay” may be absent if feasible technique is not available. A comparison of the methods is listed in Table 4. The numbers of different analytical methods applied in “Assay” section for herbal drugs in both EP and ChP are shown in Fig. 1. It is shown that HPLC remains the most applied analytical method in TCM assay, followed by essential oil determination, ultraviolet–visible spectroscopy (UV–Vis) and GC. HPLC in TCM assay has many advantages, such as high separation efficiency, wide range of application, good reproducibility, accuracy and short analysis time. Thus, HPLC is the most preferred techniques in TCM assay. Among the 73 TCM reviewed, 57 in EP and 60 in ChP employ HPLC for “Assay”. While for volatile components determination, GC possesses many advantages over LC and therefore is used more often in herbal drugs with high content of volatile compounds. For example, Amomi Fructus, Amomi Fructus Rotundus, Foeniculi Fructus and Anisi Stellati Fructus all include GC analysis for “Assay” in both pharmacopoeias. Besides LC and GC, UV–Vis is another quantification technique used to determine a specific group of components with high degree of conjugation or can be highly conjugated after derivatization. Among the “Assay” of the 73 TCM monographs, 6 in EP and 5 in ChP apply UV–Vis method to determine flavonoids, alkaloids, tannins, etc. in the herbal drugs, showing that although many active components determination has been done by LC and GC, UV–Vis spectroscopy is still useful in quality control of TCMs, especially for TCMs without applicable chromatographic analysis.

**Table 4:** Comparison of herbal drugs with different methods for “Assay” in European Pharmacopoeia and Chinese Pharmacopoeia

European Pharmacopoeia			Chinese Pharmacopoeia		
Latin Name	Method	Limit	Latin name	Method	Limit
Traditional Chinese medicines in both EP and ChP					
Angelicae Sinensis Radix	LC	*trans*-Ferulic acid (≥ 0.050%)	Angelicae Sinensis Radix	SD HPLC	Essential oil (≥ 0.4%) Ferulic acid (≥ 0.050%)
Atractylodis Lanceae Rhizoma	SD	Essential oil (≥ 14 mL/kg)	Atractylodis Rhizoma	HPLC	Atractylodin (≥ 0.30%)
Atractylodis Macrocephalae Rhizoma	SD	Essential oil (≥ 9 mL/kg)	Atractylodis Macrocephalae Rhizoma	Not included	
Bistortae Rhizoma	UV–Vis	Tannins (≥ 3.0%, expressed as pyrogallol)	Bistortae Rhizoma	HPLC	Gallic acid (≥ 0.12%)
Carthami Flos	UV–Vis	Total flavonoids (≥ 1.0%, expressed as hyperoside)	Carthami Flos	HPLC	Hydroxysafflor yellow A (≥ 1.0%), kaempferol (≥ 0.050%)
Clematidis Armandii Caulis	LC	Oleanolic acid (≥ 0.30%)	Clematidis Armandii Caulis	Not included	
Houttuyniae Herba	LC	Qquercitrin (≥ 0.10%)	Houttuyniae Herba	Not included	
Ligustici Chuanxiong Rhizoma	SD	Essential oil (≥ 3.5 mL/kg)	Chuanxiong Rhizoma	HPLC	Ferulic acid (≥ 0.10%)
Ligustici Rhizoma et Radix	SD	Essential oil (≥ 5.0 mL/kg)	Ligustici Rhizoma et Radix	HPLC	Ferulic acid (≥ 0.050%)
Lycii Fructus	Not included		Lycii Fructus	UV–Vis HPLC	Polysaccharide (≥ 1.8%, expressed as glucose) Betaine (≥ 0.50%)
Lycopi Herba	LC	Rosmarinic acid (≥ 0.15%)	Lycopi Herba	Not included	
Piperis Fructus	SD LC	Essential oil (≥ 25 mL/kg) Piperine (≥ 3.0%)	Piperis Fructus	HPLC	Piperine (≥ 3.3%)
Piperis Longi Fructus	SD LC	Essential oil (≥ 6.0 mL/kg) Piperine (≥ 3.0%)	Piperis Longi Fructus	HPLC	Piperine (≥ 2.5%)
Sanguisorbae Radix	UV–Vis	Tannins (≥ 5.0%, expressed as pyrogallol)	Sanguisorbae Radix	UV–Vis HPLC	Tanninoids (≥ 8.0%, expressed as gallic acid) Gallic acid (≥ 1.0%)
Uncariae Rhynchophyllae Ramulus cum Uncis	LC	Total alkaloids (≥ 0.2%, expressed as isorhynchophylline)	Uncariae Ramulus cum Uncis	Not included	
Other herbal drugs in both EP and ChP					
Aloe Barbadensis	UV–Vis	Hydroxyanthracene derivatives (≥ 28.0%, expressed as barbaloin)	Aloe	HPLC	Barbaloin (≥ 16.0%)
Aloe Capensis	UV–Vis	Hydroxyanthracene derivatives (≥ 18.0%, expressed as barbaloin)	Aloe	HPLC	Barbaloin (≥ 6.0%)
Benzoe Tonkinensis	Titration	Total acids (35.0%-55.0%, expressed as benzoic acid)	Benzoinum	HPLC	Total balsamic acid (≥ 27.0%, expressed as benzoic acid)
Caryophylli Flos	SD	Essential oil (≥ 150 mL/kg)	Caryophylli Flos	GC	Eugenol (≥ 11.0%)
Chelidonii Herba	UV–Vis	Total alkaloids (≥ 0.6%, expressed as chelidonine)	Chelidonii Herba	HPLC	Chelerythrine (≥ 0.020%)
Curcumae Longae Rhizoma	SD UV–Vis	Essential oil (≥ 25 mL/kg) Dicinnamoyl methane derivatives (≥ 2.0%, expressed as curcumin)	Curcumae Longae Rhizoma	SD HPLC	Essential oil (≥ 7.0%) Curcumin (≥ 1.0%)
Hyperici Herba	UV–Vis	Total hypericins (≥ 0.08%, expressed as hypericin)	Hyperici Perforati Herba	HPLC	Hyperoside (≥ 0.10%)
Lini Semen	Not included		Lini Semen	GC	Sum of linoleic acid and α-linolenic acid (≥ 13.0%)
Myrrha	Not included		Myrrha	SD	Essential oil (≥ 4.0% for natura myrrh, ≥ 2.0% for colloidal myrrh)
Polygalae Radix	Not included		Polygalae Radix	HPLC	Tenuifolin (≥ 2.0%), polygalaxanthone III (≥ 0.15%), 3,6′-disinapoyl sucrose (≥ 0.50%)
Polygoni Avicularis Herba	UV–Vis	Flavonoids (≥ 0.30%, expressed as hyperoside)	Polygoni Avicularis Herba	HPLC	Myricitrin (≥ 0.030%)
Rhei Radix	UV–Vis	Hydroxyanthracene derivatives (≥ 2.2%, expressed as rhein)	Rhei Radix et Rhizoma	HPLC	Total anthraquinone (≥ 1.5%, hydrolysis and expressed as sum of aloe-emodin, rhein, emodin, chrysophanol and physcion), free anthraquinone: sum of aloe-emodin, rhein, emodin, chrysophanol and physcion (≥ 0.20%)
Taraxaci Officinalis Herba cum Radice	Not included		Taraxaci Herba	HPLC	Cichoric acid (≥ 0.45%)
Trigonellae Foenugraeci Semen	Not included		Trigonellae Semen	HPLC	Trigonelline (≥ 0.45%)
Zingiberis Rhizoma	SD	Essential oil (≥ 15 mL/kg)	Zingiberis Rhizoma	SD HPLC	Essential oil (≥ 0.8%) 6-Gingerol (≥ 0.60%)

*GC* gas chromatography, *HPLC* high performance liquid chromatography, *LC* liquid chromatography, *SD* steam distillation, *UV–Vis* ultraviolet–visible spectroscopy

### Sample preparation

Sample preparation is very important in quality control of TCM because it started at the very early stage of an analysis and it has great impact on the performance of an analysis including selectivity, sensitivity and accuracy [31]. In both pharmacopoeias, heating under reflux and ultrasonication are the most used methods to extract the desired components in TCM, sample pretreatment including solid phase extraction (SPE), liquid–liquid extraction, pH adjustment, precipitation and centrifugal separation may also be applied to eliminate undesired influence of impurities, transform components into detectable compounds and enhance the extraction efficiency of the target compounds. Since quality control standard requires the method to be sensitive, stable, accurate and considerably simple and convenient, advanced sample preparation techniques may benefit the improvements of quality standard of TCM.

### Marker

In quality control of TCM, since the composition and pharmacological effects of TCM are usually very complicated, single component, multiple components or even the global chemical profile with many components may be utilized to assess the quality of a specific TCM. Chemical reference substance (CRS) and herbal reference substance (HRS) in EP or CRS, reference extract and reference crude drug in ChP could be used as reference standards for TCM identification and compound quantification. Therefore, choosing the appropriate marker is very important for accurate and valid quality assessment of TCM, especially when active markers are not available in analysis. By comparing the markers used in both pharmacopoeias, interesting differences are observed. Of the TCM reviewed, besides different choices of active markers in analysis, EP has included many analytical markers, which serve solely for analytical purposes and irrespective of any pharmacological or therapeutic activity, in TCM identification and quantification. For example, aescin and arbutin are used as analytical makers for TLC identification of Anemarrhenae Asphodeloides Rhizoma and Notoginseng Radix; caffeine is used as reference for the determination of pinoresinol diglucoside in Eucommiae Cortex. While ChP has included many reference crude drugs in TCM identification. Among the 73 TCM reviewed, 37 in ChP employ reference extract or reference drug in TCM identification, and 12 of them include only reference drug in monographs for TLC identification. In addition, EP has included many specific references for system suitability assessment, while ChP uses the intensity markers or active markers in TCM identification or assay for this purpose. For example, in EP, isoeugenol and methyleugenol are used for the system suitability test of TLC identification of Ophiopogonis Radix. Propyl parahydroxybenzoate and saikosaponin A are employed for the system suitability of LC quantification for Bupleuri Radix. Moreover, 20 out of 73 TCM apply HRS in system suitability assessment of LC assay. From the above, it is shown that other than active markers, analytical markers and HRS or reference drug are also applied in monographs either as substitution of active markers or for method validation and system suitability assessment etc., so they are important alternates when active markers are not available or with high costs.

### Prepared slices and TCM processing

In clinical applications, TCM may be processed in some ways into prepared slices based on the theory of traditional Chinese medicine. TCM processing could be as simple as washing, cleaning, cutting and smashing, to more complicated procedures such as stir-frying, steaming and treating with honey, vinegar or wine, etc. [32]. In ChP, general chapter “0213 The processing of crude drugs” specifies the relevant TCM processing methods. While for EP, although official monographs are not included, a draft general chapter “5.18 Methods of pretreatment for preparing traditional Chinese drugs: general information” has been published on Pharmaeuropa, an online EDQM publication providing public inquiries on draft EP texts. In addition, for TCM existed in both raw and processed form, ChP has included a section named “Prepared slices” at the end of the TCM monograph with the information of processing method and quality control tests, and/or a separate monograph of the processed TCM. For example, Polygoni Multiflori Radix has a separate monograph named “Polygoni Multiflori Radix Praeparata” included after the monograph of the raw drug. Not only the quality control requirements including water content, total ash, markers and their contents are different, the actions and indications are also different as well. Other examples include Astragali Radix and Rehmanniae Radix, etc. The inclusion of TCM processing in pharmacopoeia is very important because the processing of TCM can change the nature of drug, reduce toxicity, ensure safety and improve efficacy due to the change of constituents and content of active and/or toxic components before and after processing [32]. Also take Polygoni Multiflori Radix as an example, it was found that both raw and processed Polygoni Multiflori Radix exerted liver protection and toxicity, and the raw drug was more toxic than the processed drug. The hepatotoxicity may dominantly be attributed to the components of anthraquinones, and it was speculated that processing may alter the composition and contents of the toxicity related ingredients [33]. The above example demonstrates that TCM processing is a very important part in TCM application, however TCM processing may involve many different aspects in quality control such as excipients used in processing, products generated by processing and diversities in processing methods, thus more investigations should be carried out in processed TCM products.

## Comparison of other herbal drugs in EP and ChP

For some herbal medicines other than the 73 TCM discussed above, even though they share the same botanical source between the pharmacopoeias, they are considered to be traditional European herbal drugs instead of TCM as mentioned at the beginning of this review. Those herbal drugs including Ginseng Radix, Liquiritiae Radix and Rhei Radix etc. has also been well known and frequently applied in herbal remedies of China. By comparing the monographs of these herbal drugs to TCM monographs, some similarities and differences are found. Although these herbal drugs may have at least one identical botanical source in the two pharmacopoeias, inclusion of other species into the monographs are also frequently happened in ChP and sometimes in EP. Also, although the medicinal parts stated in the monographs are similar, small differences (e.g. aerial parts instead of whole plant) may be occurred. The general requirements of these herbal drugs are similar to TCM, but quality control methods may be significantly different between the two pharmacopoeias since the monographs of these herbal drugs are not drafted by Working Party on TCM, and are not on the basis of ChP. For example, for Rhei Radix, there is a chemical identification test included in EP, which is not included in ChP; while for Aloe, the chemical identification method is included in ChP but not in EP. Furthermore, different assay methods may be applied for these herbal drugs including Aloes, Caryophylli Flos, Lini Semen, Rhei Radix, etc. (Table 4 and Fig. 1). The differences in the monographs of these herbal medicines between the two pharmacopoeias may be mainly originated from the different applications and indications between Europe and China. For example, according to European Medicines Agency, Curcumae Longae Rhizoma in Europe is mainly used for gastrointestinal disorders such as feelings of fullness, slow digestion and flatulence, but in ChP it is used for the relief of pain. The above illustration implies that, quality control of herbal drugs is relevant not only to the chemical compositions but also the application habits in the region. Therefore, it is important that quality control should meet the actual application of herbal drugs in daily life in order to protect the benefits and safety of consumers.

## Advanced analytical techniques for quality standard of TCM

As shown previously, quality standard of TCM consists of origins, identification, test and assay, etc. Establishment of TCM quality standard usually include selection of quality markers, development of analytical methods, validation of the method, analysis of sufficient batches of samples and finally setting the limit requirement. In general, quality control of TCM or herbal drugs is more complicated than chemical drugs because of their complexity and different aspects in quality control. Therefore, advancement in quality control techniques is very important for TCM in order to provide valid quality control methods. With years of study, many advanced technologies have been applied in quality control of herbal drugs and some of them are proved to be effective in improving the quality control methods.

### Sample preparation techniques

Sample preparation is very important in quality control of TCM because active components in TCM is complicated and usually in a very low content. In order to enhance the extraction efficiency, eliminate matrix effects and/or reduce consumption of organic solvents, modern extraction techniques such as microwave-assisted extraction, pressurized liquid extraction (PLE) and supercritical fluid extraction have been widely used for TCM. And online coupling of sample preparation with chromatographic techniques have gained increasing attention in recent years [34]. For example, an online-SPE hyphenated with polarity switching ultra-high performance LC (UHPLC)-MS/MS method was developed for the simultaneous determination of 10 aconite alkaloids and 13 ginsenosides in Shenfu injection. The validated method had advantages of high automatic, solvent-saving, and efficiency, can be adopted as a meaningful tool for the analysis of constituents in complex matrices without tedious sample preparation procedures [35]. Another online sample preparation system was configured by hyphenating PLE with HPLC via a turbulent flow chromatography column. The crude sample was placed in a hollow guard column, which was linked to a long narrow polyetheretherketone tube and warmed in the column oven. The extraction solvent was delivered at a high flow rate to generate considerable back pressure. A turbulent flow chromatography column was incorporated to trap the small molecular components and transfer the analytes to HPLC. This system was successfully applied to the analysis of Polygalae Radix [36] and Cistanches Herba [37, 38]. Online coupling of sample preparation with chromatographic method could reduce errors generated through the process and enable automation of TCM quality control that requires minimum human labor. However, research on this kind of techniques is still relatively few, and the applicability to couple different sample preparation methods to different chromatographic methods has to be studied as well.

### TLC related techniques

In present EP and ChP monographs, TLC analysis is used mostly for TCM identification and adulterants differentiation. It is a simple, rapid method which allows the simultaneous analysis of multiple samples in parallel, but suffers from the limitations such as low separation efficiency, poor reproducibility and poor sensitivity in quantification. Nevertheless, with the development of high-performance TLC (HPTLC) and the introduction of modern instrument that can provide standardized conditions, the performance of TLC has been significantly improved. Up to date, TLC and HPTLC has been successfully applied to the quantitative analysis of active ingredients in a series of herbal drugs including Astragali Radix [39], Magnoliae Officinalis Cortex [40] and Glycyrrhizae Radix et Rhizoma [41], etc. In addition, TLC-bioautography that combines TLC separation with bioassay provides a supreme method for the screening of bioactive compounds from herbal drugs directly. It can not only show the activity of the herbal drugs but also reveal which components contribute to the activity. Due to the merits of being simple, convenient and requiring no laborious isolation, TLC-bioautography has been widely employed for screening and identification of herbal drugs components with bioactivity such as anti-microbial [42], acetylcholinesterase inhibition [43, 44], α- and β-glucosidase inhibition [45, 46], free radical scavenging and antioxidation [42, 47]. Actually, ChP has already included TLC-bioautography against 2,2-diphenyl-1-picrylhydrazyl radical for the identification of Rehmanniae Radix. Furthermore, coupling of TLC with MS or LC–MS can offer the possibility for on-line identification of the active compound, which enhances the potential of TLC in screening, identification and quantification of active constituents of herbal drugs.

### UHPLC and LC–MS

In recent LC analysis of TCM, more and more research are done by using UHPLC and LC–MS, which propose a more advanced option for more efficient, accurate and feasible quality control of herbal drugs [48, 49]. UHPLC is an advanced LC technique, which could achieve better separation and performance with shorter runtime compared to conventional HPLC, through revolutionary development in LC system especially in the pumps, column and valves, etc. [50]. At present, UHPLC has been included in both pharmacopoeia for component determination in TCM or TCM prescriptions. For example, EP has included UHPLC method for the determination of the total contents of 7 flavonoids in Typhae pollis. ChP has also included UHPLC method for multiple components determination for several Chinese patent medicines, such as Qishen Yiqi dripping pill, Fufang Danshen dripping pill and Hugan capsule. Moreover, hyphenation of LC especially UHPLC with MS, which can provide the structural information of components, could greatly enhance the efficiency and performance in qualitative and quantitative analysis of herbal drugs [48, 49]. For example, LC–MS is utilized for the determination of toosendanin in Toosendan Fructus and Meliae Cortex in ChP. Xiao et al. used UHPLC–MS to identify 131 compounds and quantify seven of them in the fruits, leaves and root barks of *Lycium barbarum* [51]. Zeng et al. employed UHPLC-triple quadrupole-MS/MS to determine 20 major constituents including salvianolic acids, tanshinones, flavonoids and triterpenes in different parts of *Salvia miltiorrhiza* [52]. Furthermore, other than active components determination, LC–MS has also been applied in analysis of toxins such as pesticide residue [53, 54] and mycotoxins [55–57]. In ChP, LC–MS has been used for the test of adonifoline, a toxic alkaloid in Senecionis Scandentis Herba. EP has included UHPLC-MS method in confirmatory test for aristolochic acid I of herbal drugs. As the promotion and popularization of UHPLC and LC–MS, they may be more and more adopted for TCM and herbal drugs in pharmacopoeias.

### Headspace (HS) GC–MS

Nowadays, HS extraction and utilization of MS are widely studied in GC analysis of herbal drugs, and coupling with solid-phase microextraction (SPME) could further enhance the performance, availability and sensitivity of GC analysis [58]. In this technique, the sample is usually heated to make the volatile compounds be transferred to gas phase and then are injected to GC (static HS) or extracted by sorbent (HS-SPME). It has simplified isolation, extraction and concentration in GC analysis into one step, which will require less samples and no organic solvents in analysis [59]. HS-GC has been used for residual solvents in both EP and ChP. However, due to its limitation in precision and accuracy, it is more applied to qualitative or relative quantitative analysis of volatile components in herb drugs. Huang et al. used HS-SPME–GC–MS to identify 46 compounds and relatively determine four major volatile components in Zingiberis Rhizoma with different drying methods [60]. Zhang et al. compared the composition and relative contents of the volatile compounds in crude and processed Atractylodis Macrocephalae Rhizoma using static HS-GC–MS [61]. Chen et al. separated and relatively quantified 63 volatile compounds, with 53 being identified from three *Dendrobium* spp. samples by HS-GC–MS [62].

### Quantitative analysis of multi-components by single marker (QAMS)

It is known that the efficacy of TCM is contributed by their multi-components or in their combinations. Thus multi-components determination has been commonly accepted as the effective way for the quality control of TCM. But the major obstacles of the approach are the lack of commercial available CRS and the high costs involved. In order to resolve the problem, QAMS method that could accurately determine the contents of multiple constituents by using a single compound has been proposed. It uses a commercially available and cheap CRS as the internal standard, then the peaks of other compounds could be identified by relative retention time and the contents could be calculated by the validated relative correction factor [63]. QAMS method has been adopted in several monographs in both pharmacopoeias, such as Andrographis Herba, Aucklandiae Radix and Evodiae Fructus in EP, as well as Andrographis Herba, Coptidis Rhizoma and Salviae Miltiorrhizae Radix et Rhizoma in ChP. It has also been widely used for multi-components quantification of TCM, including Scutellariae Radix [64], Astragali Radix [65], Gastrodiae Rhizoma [66], etc. QAMS is a simple and practical method for simultaneous determination of multi-components in herbal drugs, which is expected to be utilized more widely by pharmacopoeias in the future.

### Fingerprint

Fingerprints are becoming more and more important in quality control especially in authentication and identification of TCM because of their complexity. Using fingerprint could reflect integral characterization of herbal drugs and offer the possibility for TCM quality control in a holistic manner, which is consistent with the principle of traditional Chinese medicine [67]. Hence, EP has already included some GC “chromatographic profile” for essential oil monographs such as Cassia oil, Clove oil and Eucalyptus oil. ChP has included “characteristic chromatogram” or “fingerprint” tests in some TCM and TCM’s oil, fat and extractives as well, e.g. Aquilariae Lignum Resinatum, Dendrobii Caulis, Gastrodiae Rhizoma, Notopterygii Rhizoma et Radix, Lonicerae Japonicae Flos, Notoginseng Total Saponins, and Salvia Total Phenolic Acids. Hong Kong Chinese Materia Medica Standards has also included HPLC fingerprint for TCM authentication, and a typical chromatogram is presented in the monograph of each TCM. Nowadays, fingerprint could be generated by various analytical techniques including TLC, HPLC/UHPLC, GC, infrared spectroscopy, nuclear magnetic resonance spectroscopy, etc. The combination of chemometric methods such as similarity analysis, principal component analysis and hierarchical cluster analysis can make full use of the component information of fingerprints, which is beneficial to the overall quality control of TCM. Lu et al. established HPLC fingerprint coupled with similarity, hierarchical clustering, and principal component analyses to evaluate the quality of raw and processed Corydalis Rhizoma from different origins [68]. Huang et al. used GC–MS fingerprint combined with chemometric approaches for the discrimination of Schisandrae Fructus from different species and different growing places [69]. Since techniques in fingerprint establishment are becoming more and more mature and easily assessable, inclusion of fingerprint in pharmacopoeia monographs would be more and more often and necessary for better quality control of herbal drugs.

### Molecular DNA barcoding

Besides chemical-based TCM identification, molecular identification that uses specific fragments of DNA as markers is another effective method for authentication and identification of herbal drugs. In ChP, molecular identification has been used for the identification of Dendrobii Caulis, Fritillariae Cirrhosae Bulbus, Agkistrodon, Bungarus Parvus and Zaocys. Among various molecular identification techniques, molecular DNA barcoding has been increasingly studied recently [70, 71]. The core of this advanced technique is to assess the sequence variations of one or several commonly recognized, relatively short DNA sequences in the genome of the samples for the identification and authentication of herbal drugs. The process in general could be divided into three steps: DNA extraction, polymerase chain reaction amplification and DNA sequencing. Then the data are compared, aligned and analyzed to identify and authenticate herbal drugs from adulterants. As DNA barcoding technology is more assessable nowadays, ChP has incorporated a general chapter “9107 Guidelines for molecular DNA barcoding of Chinese materia medica”, which provides information and requirements for using DNA barcoding in TCM identification. In recent years, DNA barcoding has been widely used for the authentication and discrimination of TCM with their adulterants, such as Scutellariae Radix [72], Astragali Radix [73], Bupleuri Radix [74], Uncariae Ramulus cum Uncis [75] and Corydalis Rhizoma [76]. Compared to chromatographic methods, DNA barcoding is a more specific technique in herbal drugs identification and is not easily affected by external factors such as climates, age, or plant part. But it may suffer from disadvantages such as false positive or negative results originating from poor DNA quality or wrong choice of DNA markers. Its application is also limited in identifying different medicinal parts, and not suitable for processed TCM because DNA degradation would severely occur in this circumstance [77]. Therefore, combination of chromatographic methods and DNA barcoding may provide comprehensive identification and quality control of TCM.

## Conclusions

In summary, quality control of TCM is very important in TCM application and the complexity of TCM promotes difficulties in quality control and quality standards establishment for TCM. But even so, working parties in EP and ChP have managed to assess TCM quality by regulating the origins, identification, quality parameters (such as moisture content and impurities) and active components contents in TCM. However, there are differences between the pharmacopoeias in Europe and China, including the source of TCM herbs, tests required for TCM, marker selection and assay methods etc. because of the different systems in quality control of TCM, and the application habits of TCM between Europe and China. Nevertheless, improvements may be made in the pharmacopoeias from the experience of the two parties. For example, although ChP (2020 edition) has removed the TCM containing aristolochic acids including Aristolochiae Herba and Aristolochiae Fructus, there are some TCM easily confused with the herbal drugs from *Aristolochia* species, such as Akebia Stem, Aucklandiae Radix, Clematidis Armandii Caulis, Stephaniae Tetrandrae Radix and Sinomenii Caulis. Including the test for aristolochic acids in these TCM as EP may be necessary and important to avoid the adulteration or misuse of aristolochic acids-containing drugs. On the other hand, in EP, HRS has been used for system suitability assessment of LC assay, it may be utilized for TLC identification as well, specially for the TCM without suitable active markers or lack of commercial available CRS. EP may also include methods of molecular identification and standards for residue of sulfur dioxide for more comprehensive quality control of TCM. Therefore, discussion about these issues and cooperation between different parties are urgently needed to improve and harmonize the quality standard of TCM. With the development in analytical techniques and quality control methods such as improvements in chromatography techniques as well as the application of molecular identification, new quality control measures are very likely to be used in the future for better quality assessment of herbal drugs.

## Supplementary information

**Additional file 1: Table S1.** Comparison of herbal drugs recorded in both European Pharmacopoeia and Chinese Pharmacopoeia.

## Data Availability

Not applicable.

## Abbreviations

ChP: Chinese Pharmacopoeia
CRS: Chemical reference substance
EDQM: European Directorate for the Quality of Medicines & Health Care
EP: European Pharmacopoeia
GC: Gas chromatography
HPLC: High performance liquid chromatography
HPTLC: High performance thin layer chromatography
HRS: Herbal reference substance
HS: Headspace
ICP: Inductively coupled plasma
LC: Liquid chromatography
MS: Mass spectrometry
PLE: Pressurized liquid extraction
QAMS: Quantitative analysis of multi-components by single marker
SPE: Solid phase extraction
SPME: Solid-phase microextraction
TCM: Traditional Chinese medicine
TLC: Thin layer chromatography
UHPLC: Ultra-high performance liquid chromatography
UV–Vis: Ultraviolet–visible spectroscopy
